# Urinary Elimination of Bile Acid Glucuronides under Severe Cholestatic Situations: Contribution of Hepatic and Renal Glucuronidation Reactions

**DOI:** 10.1155/2018/8096314

**Published:** 2018-04-11

**Authors:** Martin Perreault, Ewa Wunsch, Andrzej Białek, Jocelyn Trottier, Mélanie Verreault, Patrick Caron, Guy G. Poirier, Piotr Milkiewicz, Olivier Barbier

**Affiliations:** ^1^Laboratory of Molecular Pharmacology, CHU-Québec Research Centre and the Faculty of Pharmacy, Laval University, Quebec City, QC, Canada; ^2^Translation Medicine Group, Pomeranian Medical University, Szczecin, Poland; ^3^Proteomics Platform, Québec Genomic Center, CHU-Québec Research Centre and Faculty of Medicine, Laval University, Quebec City, QC, Canada; ^4^Liver and Internal Medicine, Medical University of Warsaw, Warsaw, Poland

## Abstract

Biliary obstruction, a severe cholestatic complication, causes accumulation of toxic bile acids (BAs) in liver cells. Glucuronidation, catalyzed by UDP-glucuronosyltransferase (UGT) enzymes, detoxifies cholestatic BAs. Using liquid chromatography coupled to tandem mass spectrometry, 11 BA glucuronide (-G) species were quantified in prebiliary and postbiliary stenting serum and urine samples from 17 patients with biliary obstruction. Stenting caused glucuronide- and fluid-specific changes in BA-G levels and BA-G/BA metabolic ratios.* In vitro* glucuronidation assays with human liver and kidney microsomes revealed that even if renal enzymes generally displayed lower *K*_*M*_ values, the two tissues shared similar glucuronidation capacities for BAs. By contrast, major differences between the two tissues were observed when four human BA-conjugating UGTs 1A3, 1A4, 2B4, and 2B7 were analyzed for mRNA and protein levels. Notably, the BA-24G producing UGT1A3 enzyme, abundant in the liver, was not detected in kidney microsomes. In conclusion, the circulating and urinary BA-G profiles are hugely impacted under severe cholestasis. The similar BA-glucuronidating abilities of hepatic and renal extracts suggest that both the liver and kidney may contribute to the urine BA-G pool.

## 1. Introduction

Bile acids (BAs) play a major role in cholesterol homeostasis. In the liver, cholesterol is efficiently converted into the primary cholic (CA) and chenodeoxycholic (CDCA) acids for subsequent secretion into intestine via the bile [1]. In the duodenum, these acids act as natural detergents to facilitate the absorption of dietary lipids, liposoluble vitamins, and cholesterol [2]. Significant proportions of CDCA and CA are then converted in the respective secondary lithocholic acid (LCA) and deoxycholic acid (DCA) by resident bacteria [2]. Both primary and secondary acids are reabsorbed and return to the liver via the portal circulation. Back in the liver, LCA and CDCA sustain additional biotransformations into the 6*α*-hydroxylated hyodeoxycholic acid (HDCA) and hyocholic acid (HCA), respectively [2, 3].

BAs are cytotoxic at elevated concentrations [4], and their accumulation in liver cells favors oxidative stress, inflammation, apoptosis, and subsequent damage to the liver parenchyma [2]. Such features are characteristic of cholestatic situations, where a reduction of the bile flow limits BA elimination from hepatocytes [5]. A reduction of BA hepatic levels is therefore an important goal for anticholestatic strategies [5].

Glucuronidation, catalyzed by members of the UDP-glucuronosyltransferase (UGT) family, is a major elimination mechanism for a variety of exogenous and endogenous molecules [6]. This enzymatic reaction consists in the transfer of the glucuronosyl group from uridine 5′-diphosphoglucuronic acid (UDPGA) to acceptor substrates. In humans, 19 functional UGTs have been categorized into three major UGT subfamilies, UGT1A, UGT2A, and UGT2B [7]. Among these enzymes, hepatic UGT1A3, 1A4, 2B4, and 2B7 isoforms have a remarkable capacity to convert BAs into glucuronide* in vitro* [8–12]. Bile acid glucuronidation involves either the 3/6-hydroxyl or 24-carboxyl groups of the BA steroid nucleus for the respective formation of ether 3/6G or acyl/ester 24G [8, 9, 11, 12]. UGT1A3 is the major enzyme for hepatic production of BA-24G, while UGT1A4, 2B4, and 2B7 are the main producers of ether glucuronides [8, 9, 11, 12].

An important consequence of glucuronidation is the introduction of an additional negative charge to the BA molecule, which allows BA-G transport by conjugate-transporters such as the multidrug resistance related proteins (MRPs) 3 and 4 that are present at the basolateral membrane of hepatocytes [4]. These transporters facilitate BA-G secretion into the blood, followed by enhanced urinary excretion. While increased BA-G urinary elimination has been reported in patients with acute cholestasis, the profile of these conjugates in both the circulation and urine has only been partially resolved [13–17]. On the other hand, the dogma that liver is the major site for glucuronidation has been challenged by the recent evidences that human kidney also has significant drug glucuronidation capacity [18]. Until now, only few investigations were performed to determine the contribution of renal UGTs to BA glucuronidation during cholestasis [19]. In the present study, 11 BA-G species have been quantified in serum and urine samples from patients with biliary obstruction obtained before and after biliary stenting. In parallel experiments, the BA-conjugating activities of microsomal extracts from human liver and kidney were compared.

## 2. Materials and Methods

### 2.1. Ethics Statement

All work has been conducted in accordance with the declaration of Helsinki (1964). This study was approved by the appropriate clinical study review boards at the CHU-Québec Research Centre, Laval University (*“Comité d'éthique de la recherche Clinique du CHUL,”* Québec City, QC, Canada: Projects #95.05.14 and #97.05.14) and Pomeranian Medical University (Bioethics Commission, Pomeranian Medical University in Szczecin, Poland: Resolution number BN-001/43/06). Experiments were conducted with the human subjects' understanding and consent. All patients had signed a written consent form before each procedure.

### 2.2. Materials

Bile acid glucuronides were obtained as described [11, 21]. Commercially available microsomes from human liver (pool of 26 men and 24 women,* #HO620*) and kidney (pool of 4 female and 4 male donors,* #H0610.R*) were purchased from Xenotech (Walkersville, MD). Human liver samples were from kidney donors as previously described (livers 2 to 8) [10, 12, 22]. Total RNA from individual human liver and kidney tissues was purchased from Ambion (Streetsville, Canada) (liver 1 and kidney 1) and Stratagene (Santa Clara, CA) (kidney 2). Total RNA from a pool of 5 human kidney donors was purchased from Ambion (Abingdon, UK). The Tri Reagent® was from Molecular Research Center Inc. (Cincinnati, OH). Protein assay reagents were obtained from Bio-Rad Laboratories Inc. (Marnes-la-Coquette, France). UGT antibodies were as previously described [10, 23, 24]. The secondary anti-rabbit IgG horse antibody was from Amersham (Oakville, Canada).

### 2.3. Patients with Biliary Obstruction

Seventeen patients (8 men and 9 women; mean age: 64 ± 10 years) with clinical and biochemical features of cholestasis were recruited as previously reported [20] (Supplementary Material 1). Diagnosis, biliary tree dilatation evidences, and biliary stenting procedures were extensively described in a previous report [20]. Stored urine was available from only 12 of these patients (6 men and 6 women) for BA-G measurement. Informed consent was obtained from each patient.

### 2.4. Bile Acid Glucuronides Measurement

Bile acid-glucuronide concentrations were determined from serum and urine samples (100 *μ*L) using liquid chromatography coupled to tandem mass spectrometry (LC-MS/MS) with an electrospray interface, as previously reported [11, 12, 21]. The chromatographic system consisted of an Alliance 2690 HPLC apparatus (Waters, Milford, MA), and the tandem mass spectrometry system was an API3200 mass spectrometer (Applied Biosystems, Concord, Canada).

### 2.5. Glucuronidation Assays

All glucuronidation assays were performed for 2 hours in the presence of 10 *μ*g microsomal proteins in a previously reported assay buffer [11, 12]. Kinetic parameters were assessed in liver and kidney samples using substrate concentrations ranging from 1 to 350 *μ*M. The enzyme kinetic model was selected as recommended [25], using the Sigma Plot 11.2 assisted by Enzyme Kinetics 1.3 program (SSI, San Jose, CA).

### 2.6. RNA Extraction, Reverse Transcription, and Real-Time PCR Analyses

Total RNA was isolated according to the Tri Reagent acid: phenol protocol as specified by the supplier (Molecular Research Center Inc.). The reverse transcription (RT) and quantitative PCR reactions were performed as previously described [11, 26]. The real-time PCR amplifications were performed using an ABI Prism 7500FAST instrument from Applied Biosystems (Foster City, CA). For each reaction, the final volume of 20 *μ*L comprised 10 *μ*L of SYBR Green PCR Mix, 2 *μ*L (4 *μ*M) of each previously reported primer [11], and 6 *μ*L of the indicated dilution of RT products. For each gene in each tissue, the amplification efficiency was tested using 2 to 5 log of cDNA produced from liver- or kidney-purified RNA and sequential dilutions of UGT cDNA constructs (0.0001 to 10 pg/*μ*l). The difference between standard curve and sample efficiency was below 10%, as recommended [27]. The amount of target genes was derived from linear regression with standard curves of these UGT constructs according to reported protocols [28]. Messenger RNA levels were calculated using the average molecular weight of one base-paired nucleotide (660 g/mol) and Avogadro's constant (6.022 × 10^23^ mol^−1^).

### 2.7. UGT Protein Determination

For Western blotting, microsomes (5 *μ*g) were size-separated by 10% SDS-polyacrylamide gels and transferred onto nitrocellulose membranes. All antibodies were used at 1 : 2,000 dilutions [10, 23, 24]. The housekeeping protein *β*-actin was used to ensure the equal protein loading in each lane. An anti-rabbit IgG horse antibody (1 : 10,000) conjugated with peroxidase (Amersham) was used as the second antibody, and the resulting immunocomplexes were visualized as described [12, 26].

Determination of UGT1A3 protein levels was also achieved using LC-MS/MS quantification according to the previously reported analytical strategy [10].

### 2.8. Data Analyses

Bile acid-glucuronide levels were calculated as mean ± standard error of the mean (SEM). BA-G concentrations did not satisfy the normal distribution according to the Shapiro-Wilk test; thus the Wilcoxon matched-pairs signed-rank test was used for statistical analyses of the response to treatment. Correlations were assessed by Spearman's rank correlation coefficient using the JMP Statistical Discovery V7.0.1 program (SAS Institute, Cary, NC).

## 3. Results

### 3.1. Biliary Stenting Modulates Circulating and Urinary BA-G Levels in Patients with Biliary Obstruction

**Figure 1:**
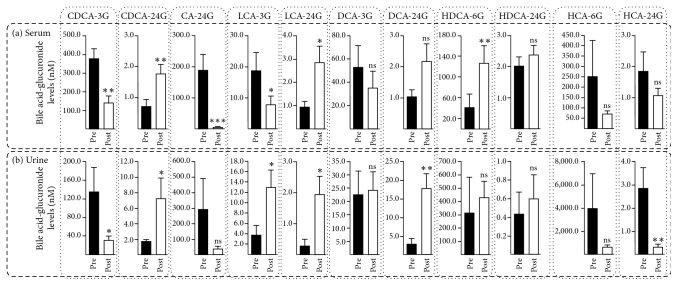
*Endoscopic stenting of the bile duct differentially affects the serum (a) and urine (b) bile acid-glucuronide profiles in patients suffering from biliary obstruction.* Serum ((a); *n* = 17) and urine ((b); *n* = 12) samples drawn before* (pre)* and after* (post)* endoscopic biliary stenting from patients with stenosed bile ducts were analyzed for their concentrations in 11 bile acid-glucuronide species using LC-MS/MS. Results represent the mean concentration ± SEM and are expressed as nM. *p* values were determined by the Wilcoxon matched-pairs signed-rank test. ^*∗*^*p* < 0.05; ^*∗∗*^*p* < 0.01; ^*∗∗∗*^*p* < 0.001. ns: not significant.

The circulating and urinary BA-G profiles in prestenting and poststenting samples from biliary stenosed patients are illustrated in Figure 1. In sera, the species distribution was CDCA-3G > HCA-6G > CA-24G > DCA-3G = HDCA-6G > LCA-3G ≫ HDCA-24G = HCA-24G > DCA-24G = LCA-24G ≥ CDCA-24G. In urine, the most abundant species was HCA-6G > HDCA-6G = CA-24G > CDCA-3G > DCA-3G. As in serum, other species were minor components. Bile flow restoration caused significant reduction in serum CDCA-3G (2.7-fold), CA-24G (25-fold), and LCA-3G (2.4-fold) contents (Figure 1(a)). By contrast, CDCA-24G (2.4-fold), LCA-24G (3.0-fold), and HDCA-6G (3.0-fold) concentrations were significantly increased in poststenting versus prestenting sera (Figure 1(a)). Other changes failed to reach the statistical significance. As we previously reported [11], serum levels of BA-G in noncholestatic volunteers varied from 0.7 ± 0.1 nM for HDCA-24G to 63.5 ± 8.6 nM for HCA-6G. As observed in the present study, the most abundant species were HCA-6G, CDCA-3G, DCA-3G, and HDCA-6G. Biliary stenting also changed urine BA-G levels (Figure 1(b)): CDCA-24G (4.1-fold), LCA-3G (3.5-fold), LCA-24G (6.5-fold), and DCA-24G (5.9-fold) were increased; and CDCA-3G (4.3-fold) and HCA-24G (8.3-fold) were reduced.

### 3.2. Biliary Stenting Differentially Affects the BA-G versus BA Metabolic Ratios in Serum and Urine from Biliary Stenosed Patients

**Figure 2:**
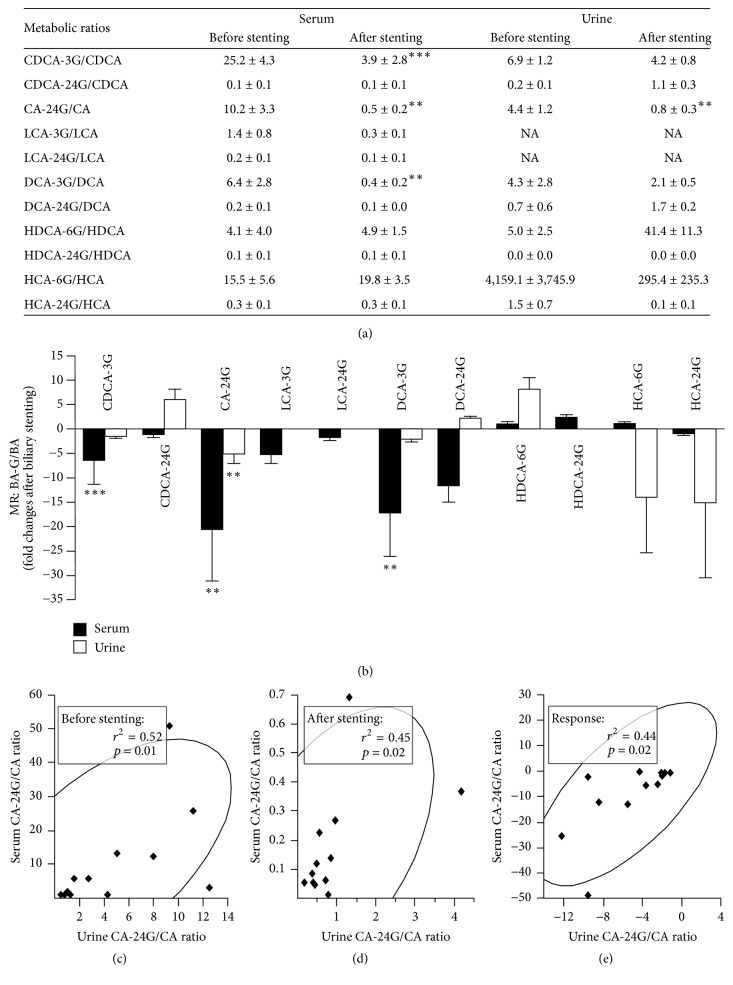
*While bile flow restoration modulates metabolic ratios of bile acid glucuronides in a glucuronide- and fluid-dependent manner ((a) and (b)), the CA-24G/CA ratio is unique in being significantly associated with serum and urine (c–e).* Serum and urine samples drawn before and after endoscopic biliary stenting from patients with stenosed bile ducts were analyzed for their concentrations in 11 bile acid-glucuronide species using LC-MS/MS. Concentrations of unconjugated acids were determined as reported [20], and the metabolic ratio for each species (a) was calculated as the ratio of glucuronide versus unconjugated precursor. (b) Black (serum; *n* = 17) and white (urine; *n* = 12) bars represent the mean ± SEM of fold changes (posttreatment values divided by pretreatment values) in MRs. Statistically significant differences between prestenting versus poststenting samples were determined using the Wilcoxon matched-pairs signed-rank test: ^*∗∗*^*p* < 0.01; ^*∗∗∗*^*p* < 0.001. Other changes failed to reach statistical significance. The absence of unconjugated LCA in urine impaired such analysis for LCA-3 and -24G [20], while urine HDCA-24G/HDCA was null. (c–e) Correlation analyses of paired (*n* = 12) prestenting (c) and poststenting (d) serum and urine CA-24G/CA values or response to stenting determined as the difference between post- and pretreatment levels (e) were performed using the Spearman rank-order correlation (*r*^2^), and *p* values for comparisons are indicated.

The serum and urine samples were previously analyzed for their content in the 6 unconjugated precursors of the glucuronide derivatives analyzed here [20]. These concentrations were used to calculate the metabolic ratio (i.e., BA-G/BA) for each glucuronide (Figure 2 and Supplementary Material 2). This parameter translates the glucuronide production capability from the available pool of unconjugated precursor, thus ensuring that changes in blood or urine levels of BA-G species do not only reflect changes in their precursors [11]. In serum samples, only the 6.5-, 20.6-, and 17.2-fold reductions in the respective CDCA-3G/CDCA, CA-24G/CA, and DCA-3G/DCA ratios were statistically significant (Figures 2(a) and 2(b)). Other changes, even the reduction in DCA-24G/DCA (11.7-fold) and LCA-3G/LCA (5.3-fold) or the 2.5-fold increase of the HDCA-24G/HDCA ratio, failed to reach statistical significance (Figure 2(b)). In urine (Supplementary Material 2), the unique significant change corresponded to the 5.2-fold reduction of the CA-24G/CA ratio (Figure 2(b)), while the 14- and 15-fold reductions in HCA-6 and -24G MRs also failed to reach statistical significance.

### 3.3. Serum and Urine BA-G Profiles Are Only Feebly Associated

Correlation studies of circulating and urinary BA-G levels in paired samples identified only few significant associations (Table 1). In prestenting samples, only the serum DCA-3G was significantly and positively correlated (*r*^2^ = 0.82) to its urinary concentration, while in poststenting fluids, no correlation for this conjugate was detected. Serum and urine CA-24G (*r*^2^ = 0.51) and LCA-24G (*r*^2^ = 0.47) levels were significantly correlated but only in samples obtained after bile flow restoration (Table 1). When the response to stenting (determined as the difference between post- and prestenting values) was analyzed, only changes for LCA-24G (*r*^2^ = 0.42), DCA-24G (*r*^2^ = 0.58), HDCA-6G (*r*^2^ = 0.35), and HCA-6G (*r*^2^ = 0.67) were positively and significantly associated (Table 1). Finally, when similar analyses were performed for MRs, the unique significant association in prestenting (*r*^2^ = 0.52) and poststenting (*r*^2^ = 0.45) levels and response (*r*^2^ = 0.44) was obtained with the ratio CA-24G/CA (Figures 2(c)–2(e)).

### 3.4. Bile Acid Glucuronidation by Human Liver and Kidney Extracts

**Figure 3:**
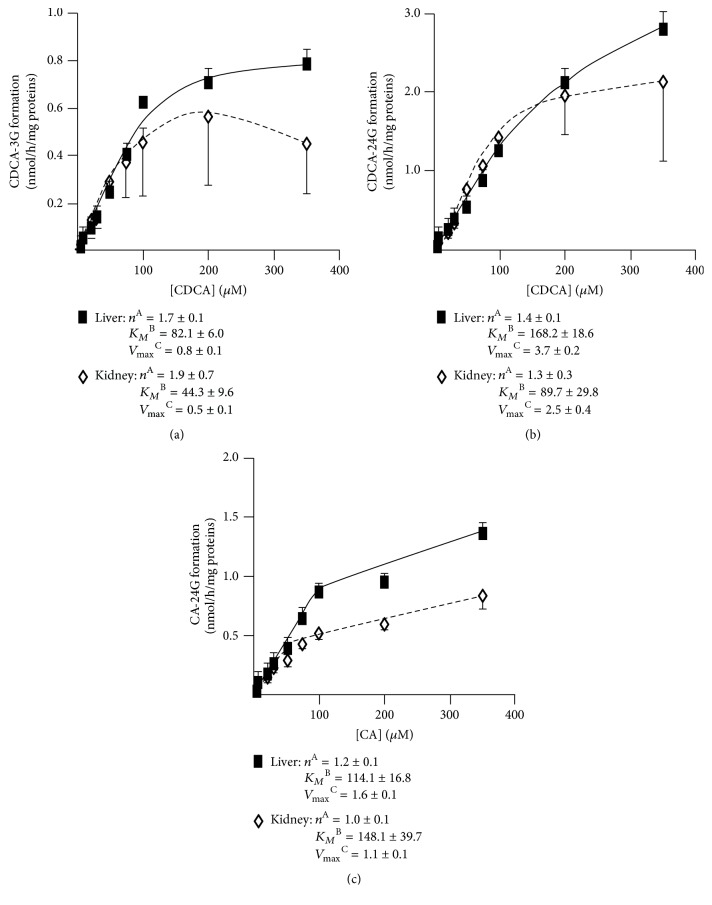
*Kinetic analyses of CDCA-3G (a), CDCA-24G (b), and CA-24G (c) formation by microsomes from human liver and kidney.* Human liver (black squares, pool of 50 donors) and kidney (white diamonds; pool of 8 donors) microsomes (10 *μ*g) were incubated in the presence of increasing concentrations (1 to 350 *μ*M) of chenodeoxycholic acid (CDCA, (a) and (b)) or cholic acid (CA, (c)) for 2 hours at 37°C. The formation of CDCA-3G (a), CDCA-24G (b), and CA-24G (c) was analyzed by LC-MS/MS. For each panel, graphs represent the rate of product formation (*y*-axis) versus substrate concentration (*x*-axis) of two experiments performed in triplicate. The listed kinetic parameters were determined as indicated in “Materials and Methods.” ^A^*n*: Hill coefficient; ^B^*K*_*M*_: calculated Michaelis constant expressed as *μ*M; ^C^*V*_max_: calculated maximal velocity expressed as nmol/h/mg proteins.

**Figure 4:**
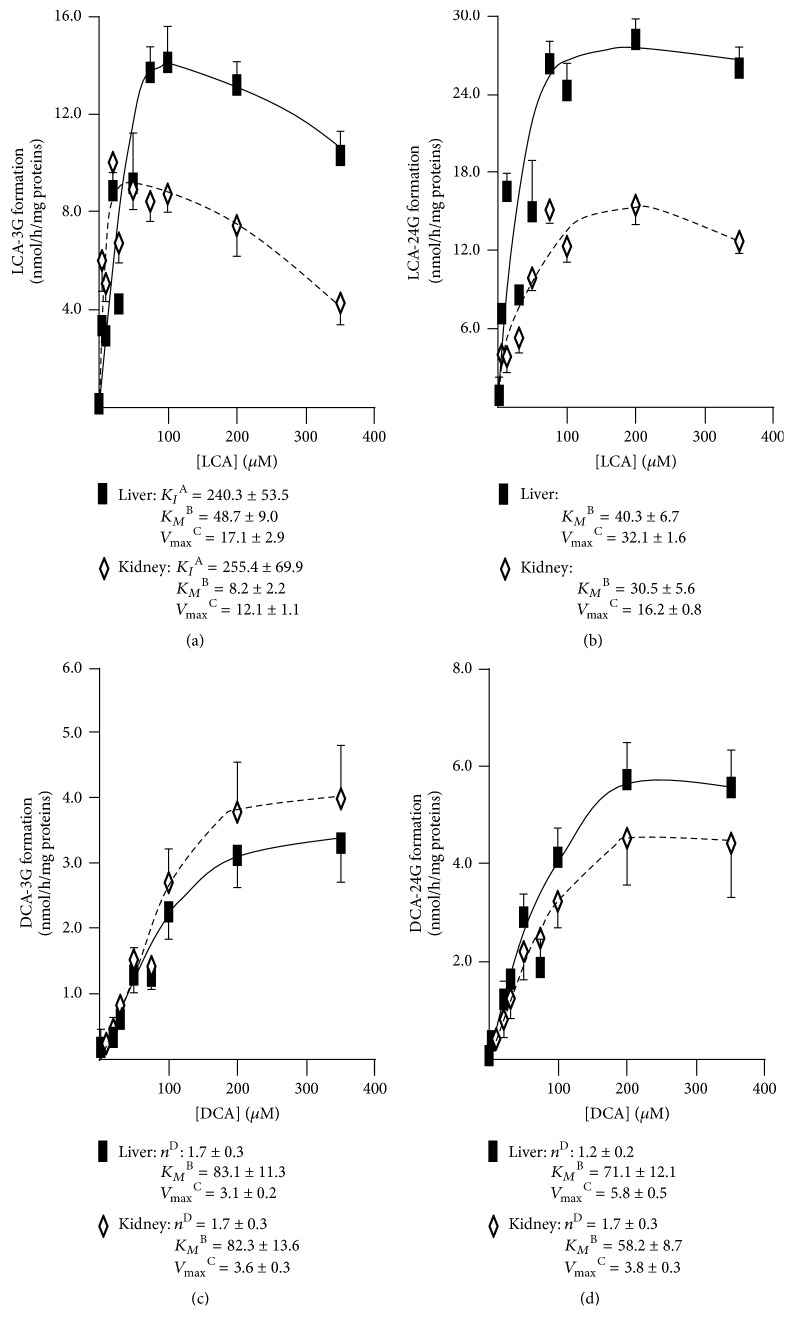
*Kinetic analyses of LCA-3G (a), LCA-24G (b), DCA-3G (c), and DCA-24G (d) formation by microsomes from human liver and kidney.* Human liver (black squares, pool of 50 donors) and kidney (white diamonds; pool of 8 donors) microsomes (10 *μ*g) were incubated in the presence of increasing concentrations (1 to 350 *μ*M) of lithocholic acid (LCA, (a) and (b)) or deoxycholic acid (DCA, (c) and (d)) for 2 hours at 37°C. The formation of LCA-3G (a), LCA-24G (b), DCA-3G (c), and DCA-24G (d) was analyzed by LC-MS/MS. For each panel, graphs represent the rate of product formation (*y*-axis) versus substrate concentration (*x*-axis) of two experiments performed in triplicate. The listed kinetic parameters were determined as indicated in “Materials and Methods.” ^A^*K*_*I*_: constant of inhibition expressed as *μ*M; ^B^*K*_*M*_: calculated Michaelis constant expressed as *μ*M; ^C^*V*_max_: calculated maximal velocity expressed as nmol/h/mg proteins; ^D^*n*: Hill coefficient.

**Figure 5:**
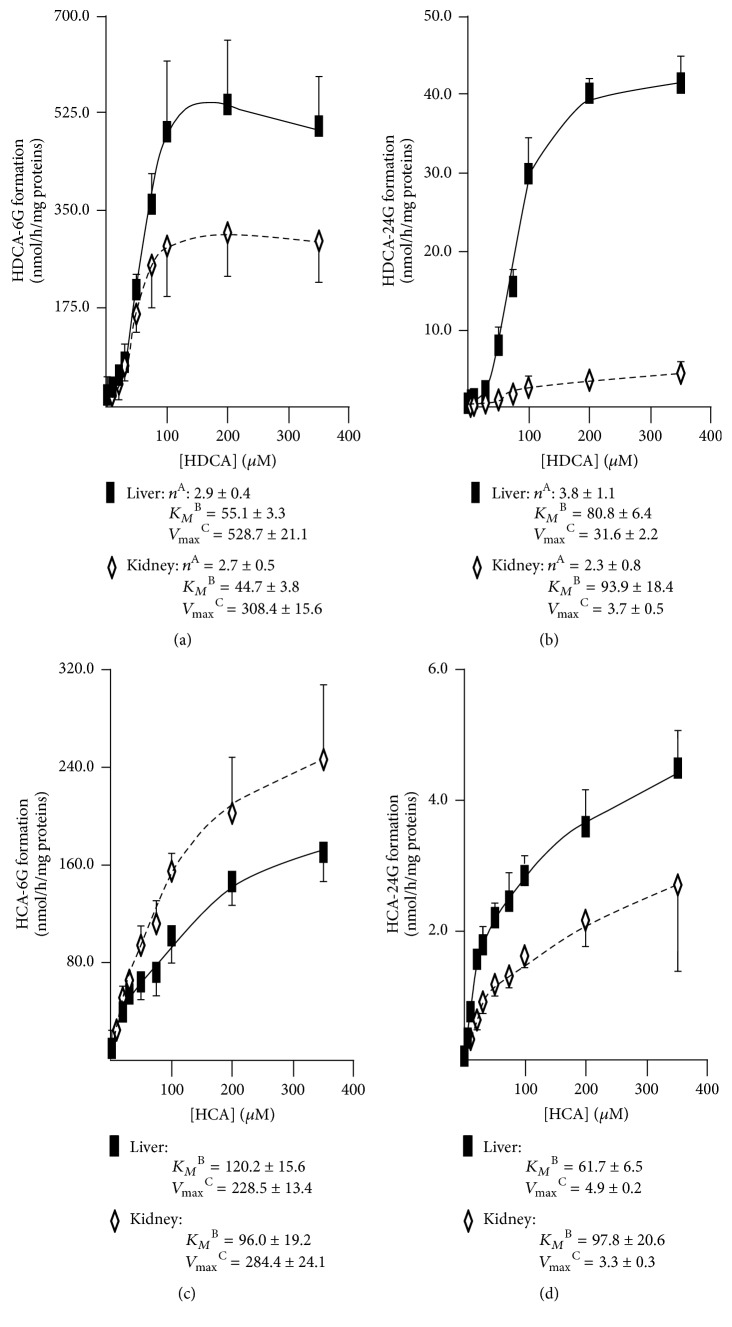
*Kinetic analyses of HDCA-6G (a), HDCA-24G (b), HCA-6G (c), and HCA-24G (d) formation by microsomes from human liver and kidney.* Human liver (black squares, pool of 50 donors) and kidney (white diamonds; pool of 8 donors) microsomes (10 *μ*g) were incubated in the presence of increasing concentrations (1 to 350 *μ*M) of hyodeoxycholic acid (HDCA, (a) and (b)) or hyocholic acid (HCA, (c) and (d)) for 2 hours at 37°C. The formation of HDCA-6G (a), HDCA-24G (b), HCA-6G (c), and HCA-24G (d) was analyzed by LC-MS/MS. For each panel, graphs represent the rate of product formation (*y*-axis) versus substrate concentration (*x*-axis) of two experiments performed in triplicate. The listed kinetic parameters were determined as indicated in “Materials and Methods.” ^A^*n*: Hill coefficient; ^B^*K*_*M*_: calculated Michaelis constant expressed as *μ*M; ^C^*V*_max_: calculated maximal velocity expressed as nmol/h/mg proteins.

The limited associations between circulating and urinary profiles observed above suggest a potential extrahepatic origin for urinary levels of BA-G. To test such a hypothesis, we sought to compare the kinetic parameters of BA glucuronidation by human liver (pool of 50 donors) and kidney (pool of 8 donors) microsomes (Figures 3–5). No differences in kinetic models were observed, with the BA-specific glucuronidation reactions being distributed among 3 models:

(i) The sigmoidal pattern for CA-24G and CDCA-24 and -3G (Figure 3), DCA-3 and -24G (Figures 4(c) and 4(d)), and HDCA-6 and -24G (Figures 5(a) and 5(b)) production

(ii) The Michaelis-Menten model for the formation of LCA-24G (Figure 4(b)) and HCA-6 and -24G (Figures 5(c) and 5(d))

(iii) The substrate-inhibition model for LCA-3G with respective *K*_*I*_ values of 240.3 ± 53.5 and 255.4 ± 69.9 *μ*M for liver and kidney extracts (Figure 4(a)).

With the exception of CA-24G, HDCA-24G, and HCA-24G, kidney UGTs generally displayed lower *K*_*M*_ values. For example, kidney microsomes exhibited a 6-fold lower *K*_*M*_ value than liver ones for LCA-3G production (Figure 4(a)). Another notable difference between liver and kidney microsomes relates to the 10-fold higher *V*_max_ value of HDCA-24G production obtained with the hepatic microsomal preparation (Figure 5(b)). Nevertheless, the most efficient reactions were obtained with the conversion of HDCA and HCA into 6G derivatives that occurred at high velocities (i.e., *V*_max_) with both liver and kidney microsomes (Figures 5(a) and 5(c)). In both tissue extracts, the 6*α*-hydroxylated acids were more actively converted into 6-glucuronide when compared to their 24-glucuronide counterparts (Figure 5). Interestingly, the opposite was observed for CDCA (Figures 3(a) and 3(b)) and LCA (Figures 4(a) and 4(b)). The *V*_max_ values for DCA-3 and -24G formation were, however, similar (Figures 4(c) and 4(d)).

### 3.5. Differential BA-Conjugating UGT Expression in the Human Liver and Kidney

**Figure 6:**
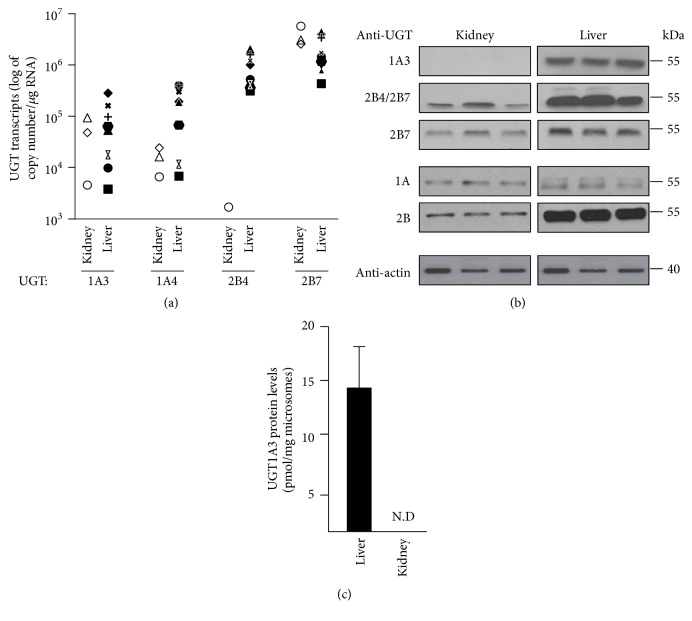
*Differential expression of the bile acid-conjugating UGT enzymes in the human liver and kidney.* (a) UGTs 1A3, 1A4, 2B4, and 2B7 mRNA levels in total RNA samples from human kidney (1 pool of 5 donors and 2 individual donors) and liver (8 individual donors) were determined through qRT-PCR analyses as described in “Materials and Methods.” Results are expressed as log_10_ of mRNA copy number per *μ*g of total RNA. Each symbol represents 1 donor, with the exception of the white triangle that corresponds to a pool of the 5 kidney donors. (b) The UGT protein contents in microsomal preparations (5 *μ*g) from human kidney (pool of 4 female and 4 male donors) and liver (pool of 26 male and 24 female donors) were determined after size separation on SDS-PAGE and hybridization with the anti-UGT1A3, anti-UGT2B4/7, anti-UGT2B7, anti-UGT1A, and anti-UGT2B antibodies (1 : 2,000 dilution). The housekeeping protein *β*-actin was used to ensure the equal protein loading in each lane. Data presented are representative of two experiments. (c) The UGT1A3 protein content in human liver and kidney microsomes was determined using LC-MS/MS analyses as previously described [10]. Data represent the mean ± SD of two different experiments performed in triplicate. N.D: not detected.

Results from kinetic experiments indicate that the human kidney possesses an efficient BA-conjugating UGT system. We next investigated whether the BA-conjugating UGTs 1A3, 1A4, 2B4, and 2B7 [8–11] are differentially expressed in human liver and kidney (Figure 6). With the exception of UGT2B4 transcripts that were detected only in 1 kidney mRNA sample, other UGT messengers were detected in all kidney and liver preparations (Figure 6(a)). As expected [28, 29], their mRNA levels sustained a strong interindividual variability. Western blot analyses evidenced, however, major differences between liver and kidney (Figure 6(b)). A clear UGT1A3 immunoreactive complex was obtained with liver extracts, while this UGT protein was not detected in microsomal proteins from kidney (Figure 6(b)). While the UGT2B7 protein was found at similar levels in both tissues, the use of an anti-UGT2B4/2B7 antibody indicated that the UGT2B4 protein may be more abundant in liver than in kidney extracts (Figure 6(b)). The lack of an accurate and specific anti-UGT1A4 antibody impaired the quantification of this BA-conjugating UGT; however, we were able to further confirm the absence of the UGT1A3 protein in kidney microsomes through the use of our previously established LC-MS/MS-based proteomic method (Figure 6(c)) [10].

## 4. Discussion

Urinary and circulating levels of BA-Gs detected in the present study are similar to previous findings in terms of both glucuronide concentrations and species distribution [13, 14, 16]. However, an important improvement of the present investigations resides in our ability to discriminate between ether and ester glucuronides of a single BA. For example, while glucuronide conjugates of CDCA, CA, and DCA were previously identified as accumulating metabolites in sera from patients undergoing bile drainage [20], the nature of these glucuronides has not been resolved until now. Our results point out the ester CDCA-3G and DCA-3G and the acyl CA-24G as the most abundant glucuronide species found in patients with biliary obstruction. Because acyl, but not ester, glucuronides can be potentially toxic molecules (reviewed in [30]), such information may be critical in anticipating the consequences of their accumulation during cholestasis.

Interestingly, serum levels of some of the bile acid glucuronides, such as CDCA-3G, CA-24G, and LCA-3G, were significantly reduced after biliary stenting, while some others, like CDCA-24G, LCA-24G, and HDCA-6G, were significantly increased. While additional investigations are warranted to decipher the mechanisms beyond such a glucuronide species-dependent responsiveness, one can speculate that these changes may actually reflect the differential manner in which the UGT enzymes and/or the BA-G transporters may be altered upon biliary obstruction and after stenting. Therefore, it would have been of interest to compare how these proteins behave in tissues (i.e., liver and kidney) from cholestatic donors to validate such a hypothesis. Actually, one limitation of the current study is that the liver and kidney microsomes used are not from donors with biliary obstruction. When compared to other glucuronide conjugates, CA-24G presented a unique behavior: (1) in contrast to other acyl BA-Gs that are only minor components of the urine and serum glucuronide pools, CA-24G is the 3rd most abundant glucuronidated acid in prestenting samples; (2) this species is the most spectacularly affected when poststenting versus prestenting sera profiles are compared; (3) the serum CA-24G/CA ratio sustains the stronger reduction after biliary stenting; (4) this MR is also unique in being significantly reduced in posttreatment urines; and (5) only the CA-24G/CA ratio exhibits positive serum/urine association in terms of prestenting and poststenting distribution, as well as treatment response. These last observations support a hepatic origin for the formation of CA-24G and the strong CA-24G accumulation in prestenting fluids, and its spectacular reduction after bile flow restoration suggests that, under noncholestatic situations, this compound, formed in liver cells, is normally secreted in bile. This hypothesis is further supported by previous investigations, in which the intravenous injections of cholate glucuronide to rats resulted in a rapid and efficient secretion in bile of the unchanged glucuronide conjugate [31]. By contrast, when the same procedure was applied to bile duct ligated animals, 95% of the radioactivity was recovered in urine [31]. The present study indicates that similar rerouting of CA-24G elimination to urine may also occur in cholestatic patients, since circulating levels detected in prestenting cholestatic donors were almost 50-fold higher than those previously quantified in samples from noncholestatic volunteers (~2 nM, [11]). Privileging secretion from liver cells into the blood for subsequent urinary elimination may actually protect the liver against the accumulation of such abundant and potentially toxic glucuronide species during cholestasis. In the same vein, the improved formation of other conjugates such as CDCA-3G and DCA-3G, which we recently identified as nontoxic BAs [32], may also participate in the hepatic detoxification of their unconjugated CDCA and DCA precursors. Consistent with the above stated hypothesis, circulating MRs for these 2 conjugates (i.e., 25.2 and 6.4 for CDCA-3G and DCA-3G, resp.) are spectacularly increased when compared to those previously found in noncholestatic donors (0.6 and 0.1) [11]. However, the fact that circulating and urinary MRs for these species were not associated and the fact that biliary stenting exerted only minor effects on these ratios in urine are also indicative of an extrahepatic origin for urine CDCA- and DCA-3G species, at least after bile flow restoration.

We next compared the human liver and kidney in terms of bile acid glucuronidation activity and BA-conjugating UGT expression. With 2 exceptions (i.e., the 6- and 10-fold difference in *K*_*M*_ and *V*_max_ values for LCA-3G and HDCA-24G formations, resp.), these experiments reveal the remarkable similarity of renal and hepatic bile acid glucuronidation processes and the clear preference of both tissues in producing HDCA-6 and HCA-6G. Such preference may actually explain the predominance of these conjugates in various human fluids as previously described [11]. However, the most intriguing observation issuing from these analyses relates to the UGT1A3 enzyme. Indeed, not only was this enzyme detected in kidney extracts only at the mRNA (but not protein) level, but also the absence of such an active enzyme, previously identified as the main isoform for acyl BA glucuronidation [8–10, 12], is inconsistent with the elevated acyl glucuronide production capability of kidney microsomes. A plausible explanation for the conflicting observation on UGT1A3 mRNA and protein expression in kidney samples relates to the fact that protein and RNA extracts were from different sources and thus were not paired. It is therefore possible that only donors for mRNA express these isoforms in the kidney. Meanwhile, unlikely, such a possibility is supported by the controversial nature of the UGT1A3 expression in the human kidney [29, 33–35]. Additional investigations are therefore warranted to clarify whether this BA-conjugating protein is found or not in the human kidney.

Nevertheless, the present study clearly establishes the major contributions that liver and kidney play in glucuronidating bile acid during cholestasis. Interestingly, Zhou and colleagues reported that intestinal glucuronidation also plays an important role in controlling the local bile acid homeostasis and the pathological development of colitis [36]. These authors elegantly demonstrated that, in the intestine, bile acids glucuronidation leads to reduced intracellular levels of ligands for the nuclear receptor FXR. Reduced FXR activation limits the secretion of FGF15/19 in the portal circulation, which in turns leads to an increase of de novo formation of bile acid in the liver [36]. Thus, in contrast to the liver and kidney, where glucuronidation is thought to facilitate the removal of bile acids, the intestinal glucuronidation seems to increase the delivery of bile acids. However, it should be noted that, during obstructive cholestasis, the delivery of hepatic bile acids to the intestine is compromised, and thus only minor intestinal glucuronidation may occur.

## 5. Conclusion

In conclusion, the present study evidences the major impact that bile flow restoration exerts on the circulating and urinary bile acid-glucuronide profiles and demonstrates the elevated capability of the human kidney to glucuronidate these endogenous compounds. Future investigations are required to clarify the mechanism allowing kidney extracts to convert bile acids into acyl glucuronides in the absence of the UGT1A3 protein.

## Figures and Tables

**Table 1:** Summary of the correlation studies between serum and urine bile acid-glucuronide profiles.

Urine levels	Before stenting	After stenting	Response
	(*r*^2^; *p* value)	(*r*^2^; *p* value)	(*r*^2^; *p* value)
CDCA-3G	0.10; 0.30	0.02; 0.67	0.25; 0.10
CDCA-24G	0.01; 0.69	0.05; 0.50	0.02; 0.63
CA-24G	0.32; 0.05	***0.51; 0.01***	0.07; 0.42
LCA-3G	0.00; 0.97	0.19; 0.15	0.01; 0.81
LCA-24G	0.14; 0.22	***0.47; 0.01***	***0.42; 0.02***
DCA-3G	***0.82; <0.0001***	0.14; 0.23	0.01; 0.71
DCA-24G	0.06; 0.43	0.16; 0.19	***0.58; 0.004***
HDCA-6G	0.29; 0.07	0.31; 0.06	***0.35; 0.04***
HDCA-24G	0.00; 1.00	0.05; 0.47	0.03; 0.59
HCA-6G	0.29; 0.07	0.15; 0.20	***0.67; 0.001***
HCA-24G	0.00; 0.83	0.27; 0.08	0.04; 0.54

Correlation analyses were performed using the Spearman rank-order correlation (*r*^2^), and *p* values for comparisons are indicated. Response is calculated as the difference between post- and prestenting values. CA: cholic acid; CDCA: chenodeoxycholic acid; DCA: deoxycholic acid; HCA: hyocholic acid; HDCA: hyodeoxycholic acid; LCA: lithocholic acid; G: glucuronide.

## Abbreviations

BA: Bile acid
BO: Biliary obstruction
CA: Cholic acid
CDCA: Chenodeoxycholic acid
DCA: Deoxycholic acid
FXR: Farnesoid X receptor
G: Glucuronide
HDCA: Hyodeoxycholic acid
HCA: Hyocholic acid
LCA: Lithocholic acid
LC-MS/MS: Liquid chromatography-tandem mass spectrometry
UDPGA: UDP-glucuronic acid
UGT: UDP-glucuronosyltransferase
MRPs: Multidrug resistance related proteins.

## Supplementary Materials

Supplementary Materials The supplementary materials present details of patient with biliary obstruction (Supplementary Material 1) and the changes in the urine profile of bile acid glucuronides and in their metabolic ratios before and after a biliary stenting procedure in patients with biliary obstruction (Supplementary Material 2). Supplementary Material 1: patients with biliary obstruction (adapted from [20]). Supplementary Material 2: changes in the urine profile of bile acid glucuronides and in their metabolic ratios before and after a biliary stenting procedure in patients with biliary obstruction (*n* = 12).
