# Supplementary material for: Urinary Elimination of Bile Acid Glucuronides under Severe Cholestatic Situations: Contribution of Hepatic and Renal Glucuronidation Reactions
Source: Can J Gastroenterol Hepatol. 2018 Apr 11;2018:8096314. doi: 10.1155/2018/8096314 (PMC5925157; doi:10.1155/2018/8096314)
Supplement: Supplementary Materials — The supplementary materials present details of patient with biliary obstruction (Supplementary Material 1) and the changes in the urine profile of bile acid glucuronides and in their metabolic ratios before and after a biliary stenting procedure in patients with biliary obstruction (Supplementary Material 2). Supplementary Material 1: patients with biliary obstruction (adapted from [20]). Supplementary Material 2: changes in the urine profile of bile acid glucuronides and in their metabolic ratios before and after a biliary stenting procedure in patients with biliary obstruction (n = 12). [file 8096314.f1.pdf]

***Supplementary material 1*. Patients with biliary obstruction (adapted from [24]).**

Seventeen patients (8 men and 9 women) with clinical and biochemical features of cholestasis were recruited [24]. In all cases, dilatation of the biliary tree was first confirmed with abdominal ultrasound. Final clinical diagnoses in patients included were as follows: common bile duct stones, 8 patients; pancreatic tumor, 5 patients; bile duct tumor, 1 patient; benign common bile duct stenosis, 1 patient and chronic pancreatitis, 1 patient. The endoscopic retrograde cholangiopancreatography (ERCP) procedure involved placement of plastic 10F stents which were removed or replaced at a median of 5 weeks after initial procedure, depending on the diagnosis.

	Normal Range	Before stenting	After stenting
AST (U/L)	5 - 43	219.67 ± 36.12	33.30 ± 6.28***
ALT (U/L)	5 - 60	290.17 ± 55.04	44.90 ± 12.03***
AP (U/L)	20 - 140	715.23 ± 189.91	157.10 ± 23.73***
γGT (U/L)	5 – 80	1154.58 ± 225.31	238.90 ± 91.74**
Total bilirubin (mg/dl)	0.1 - 1.2	15.06 ± 1.65	1.60 ± 0.32***

Liver biochemistries were determined before and after an endoscopic stenting of the bile duct. Values represent the mean ± SEM. Statistically significant differences between pre- and post-stenting samples were determined by the Wilcoxon/Mann-Whitney rank-sum test: **:$p<0.01$; ***:$p<0.001$. ALT: alanine aminotransferase; AST: aspartate aminotransferase; AP: alkaline phosphatase, γGT: γ-glutamyltranspeptidase.

Supplementary material 2. **Changes in the urine profile of bile acid-glucuronides and in their metabolic ratios before and after a biliary stenting procedure in patients with biliary obstruction (n=12).**

Urine levels	Pre-stenting (nM)	Post-stenting (nM)
CDCA-3G	135.2 ± 52.2	31.7 ± 8.3*
CDCA-24G	1.8 ± 0.2	7.3 ± 2.6*
CA-24G	293.1 ± 199.2	40.0 ± 12.6
LCA-3G	3.7 ± 1.8	13.0 ± 3.3*
LCA-24G	0.3 ± 0.2	2.0 ± 0.6**
DCA-3G	22.7 ± 8.7	24.2 ± 6.9
DCA-24G	3.0 ± 1.4	17.8 ± 3.9**
HDCA-6G	311.7 ± 271.1	431.4 ± 120.7
HDCA-24G	0.4 ± 0.2	0.6 ± 0.3
HCA-6G	3,995.1 ± 2,962.3	679.0 ± 162.2
HCA-24G	2.9 ± 0.9	0.3 ± 0.1**
Metabolic ratios		
CDCA-3G/CDCA	6.9 ± 1.2	4.2 ± 0.8
CDCA-24G/CDCA	0.2 ± 0.1	1.1 ± 0.3
CA-24G/CA	4.4 ± 1.2	0.8 ± 0.3**
LCA-3G/LCA	*NA*	*NA*
LCA-24G/LCA	*NA*	*NA*
DCA-3G/DCA	4.3 ± 2.8	2.1 ± 0.5
DCA-24G/DCA	0.7 ± 0.6	1.7 ± 0.2
HDCA-6G/HDCA	5.0 ± 2.5	41.4 ± 11.3
HDCA-24G/HDCA	0.0 ± 0.0	0.0 ± 0.0
HCA-6G/HCA	4,159.1 ±3,745.9	295.4 ± 235.3
HCA-24G/HCA	1.5 ± 0.7	0.1 ± 0.1

Bile acid-glucuronide concentrations are expressed in nM. Values represent the mean ± SEM. The metabolic ratio for each species was calculated as the ratio of glucuronide *versus* unconjugated precursor. Statistically significant differences between pre- and post-stenting

samples were determined by the Wilcoxon matched-pairs signed-ranks test: *:$p<0.05$; **:$p<0.01$. CA: cholic acid; CDCA: chenodeoxycholic acid; DCA: deoxycholic acid; HCA: hyocholic acid; HDCA: hyodeoxycholic acid; LCA: lithocholic acid; G: glucuronide. *NA*: Not applicable due to the previously reported absence of unconjugated LCA in these urine samples [24].
